# A Sustainable Synthetic Approach to Tacrine and Cholinesterase Inhibitors in Deep Eutectic Solvents under Aerobic Conditions

**DOI:** 10.3390/molecules29061399

**Published:** 2024-03-21

**Authors:** Luciana Cicco, Filippo Maria Perna, Vito Capriati, Paola Vitale

**Affiliations:** Dipartimento di Farmacia—Scienze del Farmaco, Università degli Studi di Bari Aldo Moro, Consorzio Interuniversitario Nazionale “Metodologie e Processi Innovativi di Sintesi” (C.I.N.M.P.I.S.), Via E. Orabona 4, I-70125 Bari, Italy; filippo.perna@uniba.it (F.M.P.); paola.vitale@uniba.it (P.V.)

**Keywords:** tacrine, cholinesterase inhibitors, deep eutectic solvents, green chemistry, azides

## Abstract

An enhanced, sustainable, and efficient method for synthesizing tacrine, achieving a 98% yield, has been developed by replacing volatile organic compounds with more eco-friendly solvents such as deep eutectic solvent (DESs). The optimized protocol scales easily to 3 g of substrate without yield loss and extends successfully to tacrine derivatives with reduced hepatotoxicity. Particularly notable is the synthesis of novel triazole-based derivatives, yielding 90–95%, by integrating an in situ preparation of aryl azides in DESs with *N*-propargyl-substituted tacrine derivatives. Quantitative metrics validate the green aspects of the reported drug development processes.

## 1. Introduction

In a world with dwindling petroleum resources, researchers are continuously nurturing the development of safer methodologies and waste prevention as a response to the growing demand for sustainable and environmentally friendly processes with a low carbon footprint. In particular, owing to the heavy impact of solvents on pollution and the organic waste produced (over 80%), green and sustainable solvents are progressively replacing volatile organic compounds (VOCs) in several chemical and extraction processes both in academia and industry [1,2]. Deep eutectic solvents (DESs) are among the latest breakthroughs in the realm of green solvents due to their low volatility, nonflammability, and tunability properties. Additionally, the remarkably low toxicity of some of them (particularly those derived from bio-based components) aligns well with their suitability for use in the pharmaceutical field [3,4,5,6]. They are usually obtained by the combination of safe, inexpensive, and nature-inspired components (Brønsted or Lewis acids and bases, anionic and/or cationic species) that, when mixed in a proper molar ratio and heated, give rise to eutectic mixtures with a eutectic point temperature lower to that of an ideal liquid mixture [7].

Nitrogen-containing heterocyclic compounds are a valuable source of therapeutic agents in medicinal chemistry. Thanks to their broad chemical structure, they can play the role of “spacers” and/or bioisosteres of various functional groups, often interacting effectively with receptors, enzymes, and biological targets [8]. DESs have been widely used in organic synthesis [9], in particular for the preparation of *N*-containing heterocyclic scaffolds because of their dual solvent–catalyst role [10,11,12]. Over the last decade, valuable sustainable methodologies have been introduced in the literature, both by our group and others, for the synthesis of pharmacologically active heterocycles with central nervous system activity or anti-inflammatory or antiproliferative properties (e.g., functionalized triazoles, pyrimidines, imidazoles, pyrazones, benzoxazines, tetrahydrofuran, and tetrahydropyran derivatives) [13,14,15,16,17,18] and of Active Pharmaceutical Ingredients (APIs) like COX-1 inhibitors [19] and antihistamine drugs (e.g., thenfadyl and some analogs, such as dimethindene) [20,21].

**Scheme 1:**
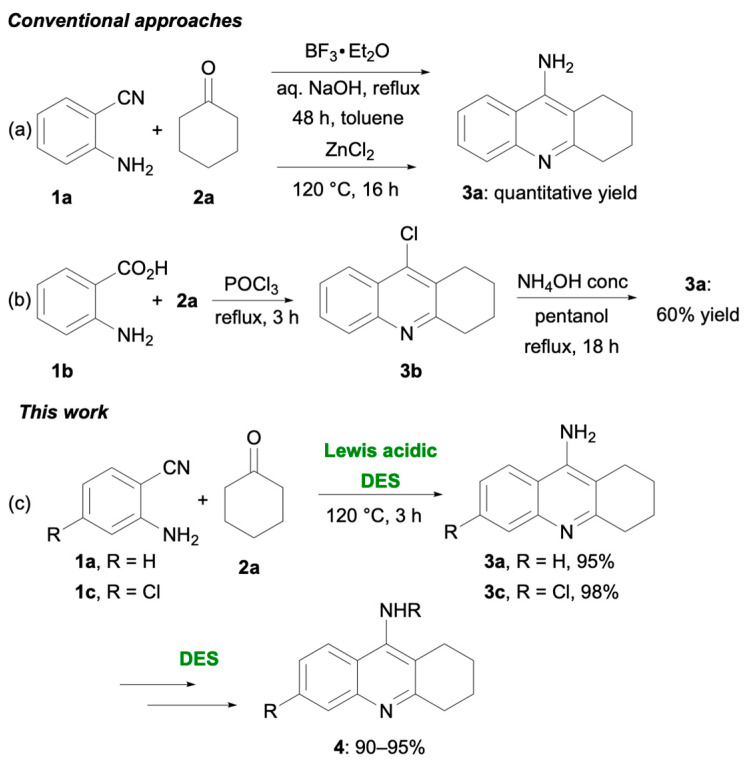
Synthetic pathways for tacrine **3a** and its derivatives (**3a**,**c** and **4**) via Friedländer annulation reaction in VOCs (**a**,**b**) and (Lewis acidic) DESs (**c**).

Tacrine is a cholinesterase inhibitor that increases the synaptic levels of acetylcholine in the treatment of neurological disorders. Moreover, it was also found to act as an anticancer inhibitor of topoisomerases and DNA transcription [22]. The preparation of tacrine derivatives with reduced toxicity (e.g., by modification of the heterocyclic ring or the chemical structure or by increasing or decreasing the ring size with new functional groups) is currently an active research area. Conventional approaches to synthesize tacrine (**3a**) often rely on intermolecular cyclodehydration reactions between anthranilonitrile (**1a**) (Scheme 1a) [23,24,25] or anthranilic acid [26] (**1b**) (Scheme 1b) [via 9-chlorotetrahydroacridine (**3b**)] and cyclohexanone (**2a**); however, these often use energy-intensive conditions with long reactions times (16–48 h), toxic VOCs (e.g., POCl_3_, toluene), and laborious work-up procedures based on VOCs (e.g., CHCl_3_, Et_2_O, MeOH), with **3a** being often purified by column chromatography. The reaction between **1a** and **2a** has also been documented to occur in xylene with the aid of *p*-toluensulfonic acid (PTSA), yielding 48% under reflux for 3 h [27], or utilizing a solid catalyst combination of PTSA/silica gel under microwave irradiation, achieving a 70% yield [28].

Herein, we report that the Friedländer annulation en route to quinoline skeletons of the type of **3a**,**c** can straightforwardly be realized by reacting **2a** with 2-aminobenzonitrile derivatives **1a**,**c**, using DESs as privileged reactions media, with tacrine derivatives **3a**,**c** being isolated in 95–98% yield, after short reaction times (3 h) at 120 °C, with no further purification required. In addition, the practicality of the proposed protocol was demonstrated by setting up a sustainable preparation of pharmacologically relevant *N*-substituted derivatives **4** (Scheme 1c) [29,30,31,32,33]. Typical metrics applied at First and Second Pass, according to the Chem21 Metrics Toolkit [34], have also been calculated for the synthesis of **3a** to demonstrate a significant advance in sustainability with respect to the state of the art.

## 2. Results and Discussion

Given the importance of Lewis acids (e.g., BF_3_, SnCl_2_ ZnCl_2_) in cyclodehydration processes [23,24,35], we began our investigation by studying the condensation reaction between an equimolar amount of **1a** and **2a** in some prototypical eutectic mixtures, namely choline chloride (ChCl)/glycerol (Gly) (1:2 mol mol^−1^) and ChCl/urea (1:2 mol mol^−1^) in the presence of ZnCl_2_ (20 mol%). Under these conditions, however, the desired adduct **3a** could be isolated in up to 20% yield (120 °C, 3 h) (Table 1, entries 1,2). Subsequent optimization of the eutectic mixture composition with acidic DESs (ADESs) [36] (Table 1, entries 3–6) revealed that a productive (98% yield) condensation could be achieved with either a ZnCl_2_/ChCl (1:1 mol mol^−1^) or a FeCl_3_·6H_2_O/Gly (3:1 mol mol^−1^) eutectic mixture, representative of type I and type IV Lewis acidic DESs (LADESs), respectively, each one acting as a solvent and catalyst (Table 1, entries 4,5). Of note, these reactions have proven to be faster (3 h, 120 °C) with respect to similar condensation reactions reported in the literature, which required a longer reaction time (16 h) and an excess cyclohexanone, the latter being used as a solvent and a reagent [24].

**Scheme 2:**
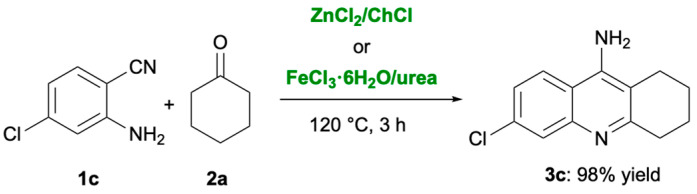
Synthesis of tacrine analogue **3c** from **1c** and **2a** in LADESs.

In order to prove the applicability of the method, we also carried out the synthesis of **3a** on a 3 g scale. The condensation reaction between **1a** and **2a** (25.4 mmol, 3 g; 25.4 mmol, 2.6 mL) in a ZnCl_2_/ChCl LADES (15 g) proceeded to uneventfully provide **3a** in 98% yield (4.9 g) as a yellow solid. The product was isolated by filtration after the addition of a 10% solution of NaOH, with no requirement for chromatography (Table 1, entry 4) (see details in the Materials and Methods Section). Similarly, the condensation between **1c** and **2a** (1 mmol each), working either in ZnCl_2_/ChCl (1:1) or in FeCl_3_·6H_2_O/urea (2:1) (1 g) LADESs, furnished tacrine derivative **3c** in a 98% yield after 3 h at 120 °C (Scheme 2).

**Scheme 3:**
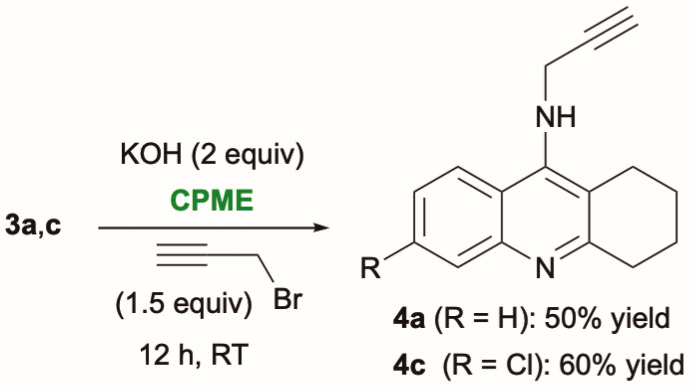
Synthesis of *N*-propargyl-substituted tacrine derivatives (**4a**,**c**) in environmentally friendly CPME.

We next focused on primary amine functionalization, as this has been proven to lead to derivatives with fewer side effects, especially in the liver district, while opening up the way for the inclusion of pharmacophores to identify a suitable molecular platform for a multitargeting approach on muscarinic agonists and antagonists. *N*-propargyl derivatives, in particular, are potent acetylcholinesterase (AChE) and butyrylcholinesterase (BChE) inhibitors [IC_50_ values: 11.2–51.3 (electric eel) and 77.6–83.5 (equine serum) nM, respectively) [37] with lower cytotoxicity and hepatotoxicity in vitro than tacrine itself [38]. After screening several DES mixtures [e.g., ChCl/Gly (1:2), ChCl/urea (1:2), ChOAc/Gly (1:2)], other environmentally friendly solvents [e.g., 2-MeTHF and ciclopentyl methyl ether (CPME)] (see ESI), and various alkaline bases [e.g., KOH, K_2_CO_3_, Cs_2_CO_3_, NaH, LiOH) (see ESI), we found that CPME [39] allowed for the isolation of derivative **4a**,**c** in 50–60% yield when treating **3a**,**c** (1 mmol) with propargyl bromide (1.5 equiv.) in the presence of KOH (2 equiv.) as the base, and when stirring the resulting mixture at room temperature (RT) for 12 h (Scheme 3).

**Scheme 4:**
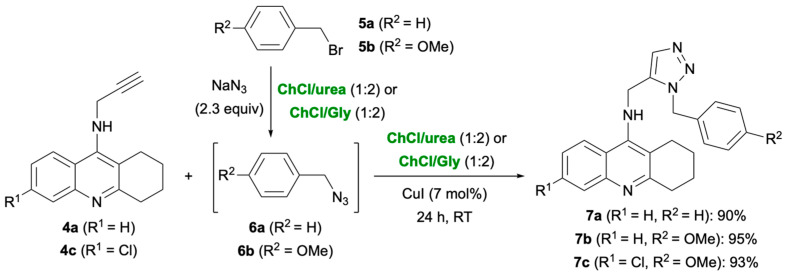
Straightforward synthesis of triazole-based tacrine derivatives **7a**–**c** by Cu-catalyzed cycloaddition reactions of *N*-propargyl-substituted derivatives **4a**,**c** and in situ-formed benzyl azides **6a**,**b** in DES.

Building on our recent achievements in the synthesis of functionalized triazoles by Cu-catalyzed cycloaddition reactions in DESs [40], we envisaged a truly green methodology to target pharmacologically relevant tacrine derivatives **7a**–**c**, which are effective for the treatment of neuro-degenerative diseases. The inhibitory activity of chloro derivative **7c**, in particular, is noteworthy, with IC_50_ values of 0.52 and 0.05 μM against AChE and BChE, respectively. Notably, while the removal of chloride resulted in a fourfold reduction in AChE inhibitory activity, compound **7b** exhibited a striking increase in BChE inhibitory activity, surpassing that of **7c** by 37-fold [41]. To this end, benzyl azides **6a**,**b** were first synthesized by reacting the corresponding benzyl bromides **5a**,**b** (1 mmol) with NaN_3_ (2.3 equiv.) in ChCl/Gly (1:2) or ChCl/urea (1:2) (1 g) (98% yield by ^1^H NMR analysis after 12 h at RT). The latter compound, without isolation, was then straightforwardly subjected to a cycloaddition reaction with *N*-propargyl-substituted tacrine derivatives **4a**,**c** (0.5 mmol) in the presence of CuI (7 mol%), thereby smoothly providing derivatives **7a**–**c** in 90–95% yield while working under aerobic conditions and vigorous stirring at RT for 24 h (Scheme 4).

To better quantify the green credentials of the synthetic pathways developed for the synthesis of tacrine and its analogs, we have calculated the Sheldon’s E-factor [42] and also have made use of some of the First and Second Pass CHEM21 Metrics Toolkit developed by Clark et al. [34], calculating atom economy (AE), reaction mass efficiency (RME), effective mass yield (EM), optimum efficiency (OE), renewable intensity (RI), renewable percentage (RP), and process mass intensity (PMI) metrics, with a breakdown of the latter for “chemicals” (reactants, reagents, and catalyst) (PMI_rxn_) and work-up and reaction solvents (PMI_WU_), and these values were compared with the corresponding ones related to the last available synthetic procedure developed in VOCs [24] (Table 2).

The classical Friedländer condensation method for the synthesis of **3a** yields an E-factor of 20 [24], whereas utilizing a ChCl-based DES reduces this value significantly to 7. An in-depth examination of the derived parameters reveals the markedly lower environmental impact of the DES-based synthetic route outlined in this study. This is particularly evident when comparing the following metrics: RME (89% in DES vs. 15% in VOCs), OE (97% in DES vs. 16% in VOCs), EM (36.3% in DES vs. 8.3% in VOCs), and RP (66.5% in DES vs. 25.2% in VOCs). It is also noteworthy that the sustainable synthetic pathway devised for tacrine synthesis eschews the use of VOCs during the isolation/purification step, which is characteristic of the traditional method employed by Dallanoce et al. [24]. This is particularly evident when comparing the PMI_WU_ values, which stand at 48.3 in VOCs versus 20.0 in DES.

## 3. Materials and Methods

### 3.1. General Methods

Deep eutectic solvents [choline chloride (ChCl)/glycerol (Gly) (1:2 mol mol^−1^); FeCl_3_·6H_2_O/urea (2:1 mol mol^−1^); ZnCl_2_/ChCl (1:1 mol mol^−1^); ChCl/urea (1:2 mol mol^−1^); FeCl_3_·6H_2_O/Gly (3:1 mol mol^−1^); and *p*-toluenesulfonic acid (PTSA)/ChCl (2:1 mol mol^−1^)] were prepared by heating under stirring the corresponding individual components at 60–80 °C for 10–30 min until a clear solution was obtained. For ^1^H NMR (600 MHz) and ^13^C NMR (150 MHz), CDCl_3_ was used as the solvent; chemical shifts are reported in parts per million (δ). FT-IR spectra were recorded on a Perkin-Elmer 681 spectrometer. GC analyses were performed on an HP 6890 model, Series II by using an HP1 column (methyl siloxane; 30 m × 0.32 mm × 0.25 μm film thickness). Analytical thin-layer chromatography (TLC) was carried out on pre-coated 0.25 mm thick plates of Kieselgel 60 F254; visualization was accomplished by UV light (254 nm) or by spraying a solution of 5% (*w*/*v*) ammonium molybdate and 0.2% (*w*/*v*) cerium(III) sulfate in 100 mL 17.6% (*w*/*v*) aq. sulphuric acid and heating to 473 K until blue spots appeared. Chromatography was run by using silica gel 60 with a particle size distribution of 40–63 μm and 230–400 ASTM. GC-MS analyses were performed on an HP 5995C model. High-resolution mass spectrometry (HRMS) analyses were performed using a Bruker microTOF QII mass spectrometer equipped with an electrospray ion source (ESI). Cyclopentyl methyl ether (CPME) was provided by Zeon Europe GmbH and was used as a solvent. Other reagents and solvents, unless otherwise specified, were purchased from Sigma-Aldrich (Sigma-Aldrich, St. Louis, MO, USA) and were used without any further purification. Full characterization data, including copies of ^1^H and ^13^C NMR spectra, have been reported for both the newly synthesized compounds and the known compounds. The following abbreviations have been used to explain the multiplicities: s = singlet, d = doublet, t = triplet, m = multiplet, br = broad, and dd = double doublet.

### 3.2. Synthesis of Tacrine ***3a*** in ZnCl_2_/ChCl (1:1 mol mol^−1^) LADES

2-Aminobenzonitrile (**1a**) (1 mmol, 118 mg) and cyclohexanone (**2a**) (1 mmol, 98 mg were added to ZnCl_2_/ChCl (1:1 mol mol^−1^) LADES (1 g). The reaction was kept at 120 °C for 3 h, then cooled to RT, and the volatiles evaporated under reduced pressure. A 10% solution of NaOH (300 µL) was added to the residue, and the mixture was stirred for an additional 3 h. After filtration, the cake was washed with water and then kept under stirring for 1 h with *i*PrOH (1 mL). The solid was filtered, and the solvent was evaporated under reduced pressure to give tacrine **3a** as a yellow solid in 98% yield (194 mg).

m.p = 178–181 °C. ^1^H NMR (600 MHz, CDCl_3_): δ 7.89 (d, *J* = 8.4 Hz, 1 H), 7.73 (d, *J* = 8.3 Hz, 1 H), 7.53–7.51 (m, 1 H), 7.33–7.28 (m, 1 H), 4.96 (br s, 2 H), 3.05–3.00 (m, 2 H), 2.59–2.48 (m, 2 H), 1.88–1.83 (m, 4 H) ppm; ^13^C NMR (150 MHz, CDCl_3_): δ 158.5, 146.6, 146.4, 128.9, 128.4, 123.8, 119.6, 117.2, 110.4, 34.1, 23.7, 22.9, 22.8 ppm; FT-IR (KBr): 3334, 3329, 2931, 2858, 1660, 1573, 1504, 1441, 1374, 1299, 976, 755 cm^−1^; GC MS (70 eV) *m*/*z* (%): 198 (M^+^, 100), 182 (22), 169 (20), 155 (5), 144 (5), 128 (6), 115 (6), 102 (7), 91 (11), 77 (9); HRMS (ESI) *m*/*z* calcd for [C_13_H_14_N_2_ + H]^+^: 199.1235, found 199.1229.

### 3.3. Synthesis of Tacrine Analogue ***3c*** in ZnCl_2_/ChCl (1:1 mol mol^−1^) or FeCl_3_·6H_2_O/urea (2:1 mol mol^−1^) LADES

4-Chloro-2-aminobenzonitrile (**1c**) (1 mmol, 152 mg) and cyclohexanone (**2a**) (1 mmol, 98 mg, were added to ZnCl_2_/ChCl (1:1 mol mol^−1^) or FeCl_3_·6H_2_O/urea (2:1 mol mol^−1^) LADES (1 g). The reaction was kept at 120 °C for 3 h, then cooled to RT, and the volatiles evaporated under reduced pressure. A 10% solution of NaOH (300 µL) was added to the residue, and the mixture was stirred for an additional 3 h. After filtration, the cake was washed with water and then kept under stirring for 1 h with *i*PrOH (1 mL). The solid was filtered and the solvent was evaporated under reduced pressure to give tacrine **3c** as a yellow solid in 98% yield (227 mg).

m.p = 260–263 °C. ^1^H NMR (600 MHz, CDCl_3_): δ 7.81 (d, *J* = 9.0 Hz, 1 H), 7.66 (s, 1 H), 7.49 (dd, 9.0 Hz, 1 H), 4.59 (br s, 2 H), 3.00 (t, *J* = 5.8 Hz, 2 H), 2.61 (t, *J* = 6.0 Hz, 2 H), 1.95–1.92 (m, 4 H) ppm; ^13^C NMR (150 MHz, CDCl_3_): δ 159.0, 145.5, 145.0, 130.6, 129.5, 129.2, 118.9, 117.9, 111.3, 34.1, 23.8, 22.8, 22.7 ppm; FT-IR (KBr): 3431, 3324, 3254, 2933, 2853, 2829, 1655, 1573, 1561, 1494, 1445, 1408, 1373, 1295, 934, 877, 823 cm^−1^; GC MS (70 eV) *m*/*z* (%): 234 (M^+^ + 2, 34), 232 (M^+^, 100), 216 (16), 204 (14), 197 (11), 180 (5), 169 (2), 152 (2), 140 (5), 128 (4), 115 (7), 108 (8), 98 (7), 91 (7), 77 (7); HRMS (ESI) *m*/*z* calcd for [C_13_H_13_ClN_2_ + H]^+^: 233.0767, found 233.0840.

### 3.4. Scale-Up Synthesis of Tacrine ***3a*** in ZnCl_2_/ChCl (1:1 mol mol^−1^) LADES

2-Aminobenzonitrile (**1a**) (25.4 mmol, 3 g) and cyclohexanone (**2a**) (25.4 mmol, 2.6 mL) were added to ZnCl_2_/ChCl (1:1 mol mol^−1^) LADES (15 g). The reaction was kept at 120 °C for 3 h, then cooled to RT, and the volatiles evaporated under reduced pressure. A 10% solution of NaOH (7 mL) was added to the residue, and the mixture was stirred for an additional 3 h. After filtration, the cake was washed with water and then kept under stirring for 1 h with 25 mL of *i*PrOH. The solid was filtered and the solvent was evaporated under reduced pressure to give tacrine **3a** as a yellow solid in 98% yield (4.9 g).

### 3.5. Representative Procedure for the Synthesis of N-Propargyl-Substituted Tacrine Derivatives ***4a**,**c*** in CPME: Synthesis of ***4a***

To a solution of tacrine **3a** (1 mmol, 198 mg), in CPME (2 mL), KOH (2 equiv., 112 mg) was added at 0 °C, and the mixture was stirred for 10 min. Then, propargyl bromide (1.5 eq., 114 µL) was added at RT, and the resulting mixture was stirred for 12 h. After the addition of 2 mL of water, the reaction mixture was extracted with CPME (3 × 1 mL). The collected organic phases were dried over anhydrous Na_2_SO_4_ and evaporated under reduced pressure. The crude was purified by column chromatography on silica gel (EtOAc as the eluent) to afford *N*-propargyl-substituted tacrine derivative **4a** as a brown solid in 50% yield (118 mg).

(**4a**) m.p = 211–213 °C. ^1^H NMR (600 MHz, CDCl_3_): δ 7.93 (dd, *J* = 14.9, 8.4 Hz, 2 H), 7.57 (t, *J* = 7.6, 1 H), 7.39 (t, *J* = 7.6, 1 H), 4.21–4.15 (m, 3 H), 3.09 (t, *J* = 6.1 Hz, 2 H), 2.82 (t, *J* = 6.0 Hz, 2 H), 2.27 (t, *J* = 2.4 Hz, 1 H), 1.95–1.89 (m, 4 H) ppm; ^13^C NMR (150 MHz, CDCl_3_): δ 159.0, 149.2, 147.4, 129.0, 128.4, 124.4, 122.3, 121.0, 118.9, 81.2, 72.5, 38.4, 34.1, 24.7, 22.9, 22.7 ppm; FT-IR (KBr): 3291, 3063, 2926, 2855, 2113, 1633, 1582, 1562, 1496, 1434, 1407, 1384, 1329, 1295, 1109, 874, 762 cm^−1^; GC MS (70 eV) *m*/*z* (%): 236 (M^+^, 89), 221 (19), 207 (100), 195 (34), 182 (44), 167 (17), 154 (9), 140 (10), 128 (9), 115 (13), 103 (14), 91 (7), 77 (14); HRMS (ESI) *m*/*z* calcd for [C_16_H_16_N_2_ + H]^+^: 237.1313, found 237.1129.

(**4c**) white solid, (162 mg, 60% yield), m.p = 125–127 °C. ^1^H NMR (600 MHz, CDCl_3_): δ 7.89 (d, *J* = 2.1 Hz, 1 H), 7.86 (d, *J* = 9.0 Hz, 1 H), 7.50 (dd, *J* = 9.0, 2.2 Hz, 1 H), 4.13 (s, 2 H), 3.06 (t, *J* = 6.0 Hz, 2 H), 2.83 (t, *J* = 5.7 Hz, 2 H), 1.94–1.89 (m, 4 H) ppm; ^13^C NMR (150 MHz, CDCl_3_): δ 159.2, 148.3, 145.7, 130.5, 130.0, 129.0, 121.7, 121.3, 119.9, 80.7, 72.5, 38.3, 33.8, 24.5, 22.6, 22.4 ppm; FT-IR (KBr): 3301, 3055, 2938, 2865, 1669, 1585, 1557, 1485, 1434, 1373, 1246, 1113, 1046, 834, 739 cm^−1^; GC MS (70 eV) *m*/*z* (%): 272 (M^+^ + 2, 6), 270 (M^+^, 17), 253 (9), 241 (32), 232 (100), 216 (20), 206 (16), 195 (15), 180 (10), 164 (8), 140 (10), 126 (8), 116 (13), 103 (11), 91 (10), 77 (9); HRMS (ESI) *m*/*z* calcd for [C_16_H_15_ClN_2_ + H]^+^: 271.0996, found 271.0991.

### 3.6. Representative Procedure for the Synthesis of Triazole-Based Tacrine Derivatives ***7a**–**c***: Synthesis of ***7a***

NaN_3_ (2.3 equiv., 82 mg) was added to a solution of benzyl bromide (0.55 mmol, 65 µL) dissolved in either ChCl/Gly (1:2 mol mol^−1^) or ChCl/urea (1:2 mol mol^−1^) (1 g). The resulting mixture was vigorously stirred at RT for 12 h. After this time, tacrine derivative **4a** (0.5 mmol) and CuI (7 mol%) were added to the reaction mixture, working under aerobic conditions and with vigorous stirring at RT. Subsequently, the mixture was allowed to stir for an additional 24 h. Upon completion of the reaction (confirmed by TLC analysis), water was added to the mixture, and the resulting aqueous phase was extracted with CPME (3 × 1 mL). The collected organic layers were dried over anhydrous Na_2_SO_4_, filtered, and the solvent was removed under reduced pressure. The crude product was finally purified by flash chromatography (EtOAc as the eluent), affording the desired product **7a** as a brown solid in 90% yield (166 mg).

(**7a**) mp 65–67 °C. ^1^H NMR (600 MHz, CDCl_3_): δ 8.00 (d, *J* = 7.8 Hz, 2 H), 7.57 (t, *J* = 7.5 Hz, 1 H), 7.37–7.34 (m, 4 H), 7.19–7.17 (m, 2 H), 5.47 (s, 2 H), 4.75 (s, 2 H), 3.05 (br s, 2 H), 2.67 (br s, 2 H), 1.85–1.82 (m, 4H) ppm; ^13^C NMR (150 MHz, CDCl_3_): δ 157.6, 150.5, 145.6, 142.2, 134.3, 129.0, 128.8, 128.6, 127.7, 127.5, 124.2, 122.6, 121.0, 120.0, 117.1, 54.0, 44.1, 33.0, 24.4, 22.5, 22.2 ppm; FT-IR (KBr): 3374, 3142, 3053, 2934, 2861, 1614, 1563, 1496, 1265, 1126, 1050, 736 cm^−1^; GC MS (70 eV) *m*/*z* (%): 369 (M^+^, 3), 197 (100), 182 (7), 91 (41), 77 (3); HRMS (ESI) *m*/*z* calcd for [C_23_H_23_N_5_ + H]^+^: 370.2032, found 370.2028.

(**7b**) white solid (189 mg, 95% yield), mp 75–78 °C. ^1^H NMR (600 MHz, CDCl_3_): δ 8.10 (dd, *J* = 17.0, 8.3 Hz, 2 H), 7.85 (t, *J* = 7.5 Hz, 1 H), 7.34 (d, *J* = 10.9 Hz, 2 H), 7.15 (d, *J* = 8.6 Hz, 2 H), 6.84 (d, *J* = 8.6 Hz, 2 H), 5.40 (s, 2 H), 4.84 (d, *J* = 4.4 Hz, 2 H), 3.77 (s, 3 H), 3.10–3.04 (m, 2 H), 2.70–2.63 (m, 2 H), 1.85–1.77 (m, 4 H) ppm; ^13^C NMR (150 MHz, CDCl_3_): δ 160.1, 156.3, 152.0, 145.3, 144.2, 129.7, 129.5, 126.4, 125.9, 124.6, 123.2, 121.3, 119.1, 116.0, 114.6, 55.3, 55.8, 44.1, 32.0, 24.4, 22.5, 22.0 ppm; FT-IR (KBr): 3399, 3128, 3061, 2921, 2850, 1612, 1586, 1563, 1514, 1251, 1210, 1032, 763 cm^−1^; GC MS (70 eV) *m*/*z* (%): 399 (M^+^, 16), 197 (100), 182 (3), 121 (54), 77 (5); HRMS (ESI) *m*/*z* calcd for [C_24_H_25_N_5_O + H]^+^: 400.2059, found 400.2140.

(**7c**) white solid (201 mg, 93% yield), mp 85–87 °C. ^1^H NMR (600 MHz, CDCl_3_): δ 7.93 (s, 1 H), 7.86 (d, *J* = 7.8 Hz, 1 H), 7.46 (d, *J* = 7.9 Hz, 1 H), 7.22 (d, *J* = 8.5 Hz, 1 H), 7.13 (d, *J* = 8.6 Hz, 2 H), 6.85 (d, *J* = 8.6 Hz, 2 H), 5.38 (s, 2 H), 4.63 (s, 2 H), 3.78 (s, 3 H), 3.00 (s, 2 H), 2.65 (s, 2 H), 1.83–1.78 (m, 4 H) ppm; ^13^C NMR (150 MHz, CDCl_3_): δ 159.9, 159.6, 159.2, 149.3, 145.6, 130.4, 129.7, 129.5, 129.2, 127.3, 126.3, 121.9, 114.5, 114.2, 113.8, 55.3, 53.7, 44.3, 29.7, 24.6, 22.7, 22.5 ppm; FT-IR (KBr): 3363, 3138, 2931, 2858, 1614, 1584, 1557 cm^−1^; GC MS (70 eV) *m*/*z* (%): 433 (M^+^, 9), 399 (12), 231 (45), 197 (75), 121 (100), 47 (25); HRMS (ESI) *m*/*z* calcd for [C_24_H_24_ClN_5_O + H]^+^: 434.1748, found 434.1712.

## 4. Conclusions

In summary, we present an enhanced, environmentally sustainable, and highly efficient method for synthesizing tacrine, achieving a remarkable 98% yield by replacing volatile organic compounds (VOCs) with environmentally responsible solvents such as Lewis acidic DESs (LADESs). Our optimized protocol demonstrates seamless scalability up to 3 g of substrate without compromising the final yield. Moreover, it proves adaptable to the synthesis of other tacrine derivatives with reduced hepatotoxicity.

Of particular significance is the synthesis of novel pharmacologically relevant triazole-based tacrine derivatives. This was achieved by integrating the in situ preparation of aryl azides in DESs with *N*-propargyl-substituted tacrine derivatives in a single reaction vessel, resulting in the isolation of the desired adducts with yields ranging from 90% to 95%. Quantitative metrics substantiate the eco-friendliness of the drug development processes detailed herein, underscoring their sustainability and environmental responsibility.

## Figures and Tables

**Table 1:** Synthesis of tacrine **3a** in eutectic mixtures ^a^.

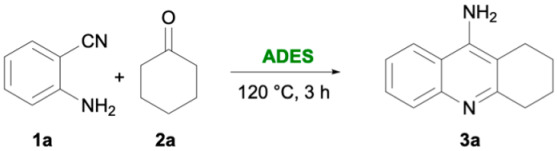		
Entry	Acidic Deep Eutectic Solvents (ADESs)	Yield 3a (%) ^b^
1	ChCl/Gly (1:2 mol mol^−1^) + ZnCl_2_ (20 mol%)	20
2	ChCl/urea (1:2 mol mol^−1^) + ZnCl_2_ (20 mol%)	18
3	FeCl_3_·6H_2_O/Gly (3:1 mol mol^−1^)	23
4	ZnCl_2_/ChCl (1:1 mol mol^−1^)	98 ^c^
5	FeCl_3_·6H_2_O/urea (2:1 mol mol^−1^)	98
6	PTSA/ChCl (2:1 mol mol^−1^)	83

^a^ General conditions: reactions performed under aerobic conditions; **1a** and **2a**: 1 mmol each; ADES: 1 g; ChCl: choline chloride; Gly: glycerol; PTSA: *p*-toluenesulfonic acid. ^b^ Isolated yield. ^c^ Reaction carried out on a 3 g scale (**1a** and **2a**: 25.4 mmol each; DES: 15 g): 98% yield of **3a**.

**Table 2:** Quantitative metrics calculated for both classical and DES-based approaches for the synthesis of tacrine **3a** ^a^.

Reaction Solvent	Yield (%)	AE (%)	RME (%)	OE (%)	EM (%)	PMI_RXN_ ^c^ (g g^−1^)	PMI_WU_ ^d^ (g g^−1^)	RI ^e^	RP (%)	E-Factor ^f^
Cy, EtOAc, MeOH ^b^	98	92	15	16	8.3	7.2	48.3	12.2	25.2	20
ZnCl_2_/ChCl ^g^	98	92	89	97	36.3	4.2	20.0	13.3	66.5	7

^a^ Each synthesis of **3a** was run on 25 mmol substrates. ^b^ Classical synthesis of **3a**, as outlined in ref. [24]; Cy: cyclohexanone. ^c^ Process mass intensity (PMI)_RXN_: chemicals and reaction solvents. ^d^ Process mass intensity (PMI)_WU_: chemicals and reaction solvents, solvents, and reagents in workup. ^e^ Renewable sources: water, DES. ^f^ This value does not consider the amount of water solution used for work-up. ^g^ ZnCl_2_/ChCl (1:1 mol mol^−1^).

## Data Availability

The data that support the findings of this study are available in the Supplementary Material of this article.

## Supplementary Materials

The following supporting information can be downloaded at: https://www.mdpi.com/article/10.3390/molecules29061399/s1, Table S1: Solvent and base screening for the synthesis of *N*-propargyl-substituted tacrine derivative **4a**; E-factor calculations; green metrics; ^1^H and ^13^C NMR spectra.
