# Supplementary material for: A Sustainable Synthetic Approach to Tacrine and Cholinesterase Inhibitors in Deep Eutectic Solvents under Aerobic Conditions
Source: Molecules. 2024 Mar 21;29(6):1399. doi: 10.3390/molecules29061399 (PMC10974547; doi:10.3390/molecules29061399)

Supporting Information

A Sustainable Synthetic Approach to Tacrine and Cholinesterase Inhibitors in Deep Eutectic Solvents under Aerobic Conditions

Luciana Cicco,* Filippo Maria Perna, Vito Capriati * and Paola Vitale

Dipartimento di Farmacia–Scienze del Farmaco, Università degli Studi di Bari "Aldo Moro", Consorzio C.I.N.M.P.I.S. Via E. Orabona 4, 70125 Bari (Italy).

Correspondence: vito.capriati@uniba.it (V.C.); luciana.cicco@uniba.it (L.C.); Tel.: +39-080-5442174 (V.C.); +39-080-5442773 (L.C.)

Table of Contents

1. Table S1. Solvent and base screening for the synthesis of *N*-propargyl-substituted Tacrine derivative **4a**. S2
2. E–factor calculations S3
3. Green metrics S5
4. ^{1}H and ^{13}C NMR spectra S10

Table S1. Solvent and base screening for the synthesis of *N*-propargyl-substituted Tacrine derivative **4a**.[a]

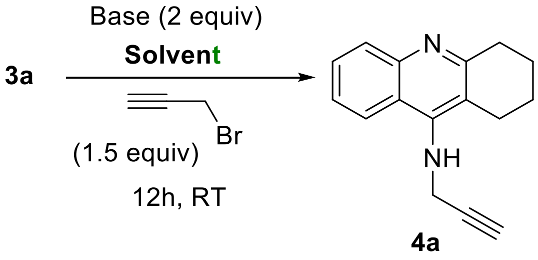

Entry	Solvent[b]	Base	Yield (%)[c]
**1**	ChCl/Gly (1:2 mol mol^{-1})	KOH	20
**2**	ChCl/Gly (1:2 mol mol^{-1})	*t*-BuOK	NR
**3**	ChCl/Gly (1:2 mol mol^{-1})	K_2CO_3	NR
**4**	ChCl/urea (1:2 mol mol^{-1})	KOH	NR
**5**	ChCl/urea (1:2 mol mol^{-1})	K_2CO_3	NR
**6**	ChCl/urea (1:2 mol mol^{-1})	Cs_2CO_3	NR
**7**	ChOAc/Gly (1:2 mol mol^{-1})	KOH	NR
**8**	2-methyltetrahydrofuran (2-MeTHF)	KOH	NR
**9**	cyclopentyl methyl ether (CPME)	KOH	50[d]
**10**	CPME	NaH	NR
**11**	CPME	LiOH	NR

[a] General conditions: reactions performed at room temperature (RT, 25 °C).; [b] solvent: 2 mL; ChCl: choline chloride; Gly: glycerol; ChOAc: choline acetate. NR = no reaction. [c] Isolated yield. [d] Reaction carried out on 1 mmol (198 mg) of **3a**, 2 equiv of base, 1.5 equiv (114 μL) of propargyl bromide, and 2 mL of CPME.

1. E–factor calculations

According to its original definition (*Green Chem.* **2007**, *9*, 1273), the Sheldon E–factor value (total mass of waste/mass of product) takes into account only the mass of waste generated in a process, and its calculation is performed by simply dividing the sum of the molecular weight of all substances produced by molecular weight of the desired products, with reference to the stoichiometric equation. Thus, the amount of silica gel, the celite pad, the drying agents, and the mass of eluent solvent used for chromatography are usually not included in the calculation. We have followed this general equation in our own calculations. Note: Note: EtOAc 0. 902 g/mL, at 25 °C; *i*PrOH 0.786 g/mL, at 25 °C; NaOH 10% 1.11 g/mL; MeOH 0.792 g/mL.

Classical synthesis of Tacrine 3a

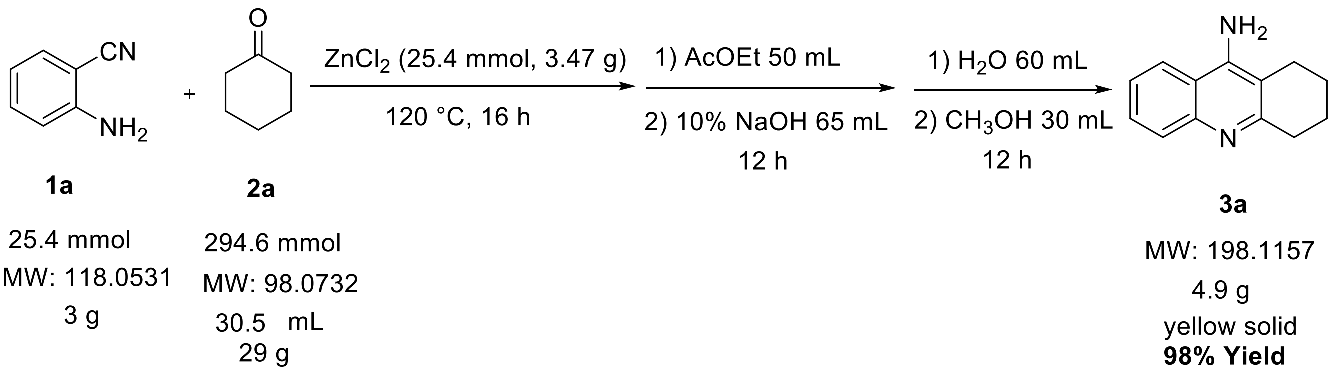

Total amount of reactants: 3 g (**1a**) + 29 g (**2a**) + 3.47 g ($ZnCl_2$) + 45.1 g (AcOEt) + 23.76 g (MeOH) = 104.33 g
Amount of the final product: 4.95 g
Amount of waste: 104.33 g – 4.95 g = 99.38 g
E–factor: amount of waste/amount of product = 99.38/4.95 = **20**

Eco–friendly synthesis of Tacrine 3a

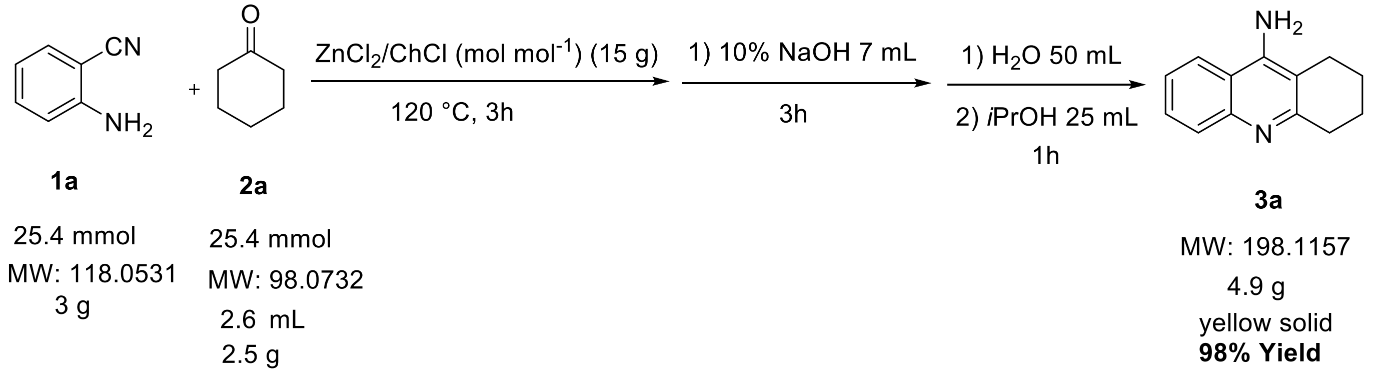

Total amount of reactants: 15 g (DES) + 3 g (**1a**) + 2.5 g (**2a**) + 20 g (*i*PrOH)= 40.5 g
Amount of the final product: 4.9 g
Amount of waste: 40.5 g – 4.9 g = 35.6 g
E–factor: amount of waste/amount of product = amount of waste/amount of product = 35.6 g/4.9 g = **7**

3. Green metrics

Metric	Acronym	Formula
Percentage yield	Y (%)	$\frac{\text{mol of product}}{\text{mol of limiting reactant}} \times 100$
Percentage conversion	Conv. (%)	$\frac{\text{final mass of limiting reactant (kg)}}{\text{initial mass of limiting reactant (kg)}} \times 100$
Percentage selectivity	Sel. (%)	$\frac{\text{\% Yield}}{\text{\% Conv.}} \times 100$
Reaction mass efficiency	RME	$\frac{\text{mass of product (kg)}}{\Sigma \text{ mass reagents (kg)}} \times 100$
Atom economy	AE	$\frac{\text{m. w product}}{\Sigma \text{ m. w. reagents}} \times 100$
Effective mass yield	EM	$\frac{\text{mass of products (kg)}}{\text{mass of non benign reagents (kg)}} \times 100$
Optimum efficiency	OE	$\frac{\text{RME}}{\text{AE}} \times 100$
Process mass intensity	PMI	$\frac{\text{total mass in a process}}{\text{mass of product}}$
Renewables intensity	RI	$\frac{\text{mass of all renewably derivable materials used}}{\text{mass of product}}$
Renewables percentage	RP	$\frac{\text{RI}}{\text{PMI}} \times 100$
Detailed solvents	*Recommended, Problematic, Hazardous, Highly Hazardous*	
Energy	• 0–70 °C *Green Flag*<br>• To 140 °C *Yellow Flag*<br>• Reflux *Red Flag*	
Workup	• *Green Flag*: quenching, filtration, centrifugation, crystallisation,<br>• *Amber Flag*: solvent exchange, quenching into aqueous solvent.<br>• *Red Flag*: chromatography, high temperature distillation	

Quantitative metrics of classical and eco–friendly approach for the synthesis of Tacrine 3a

Classical synthesis of Tacrine 3a

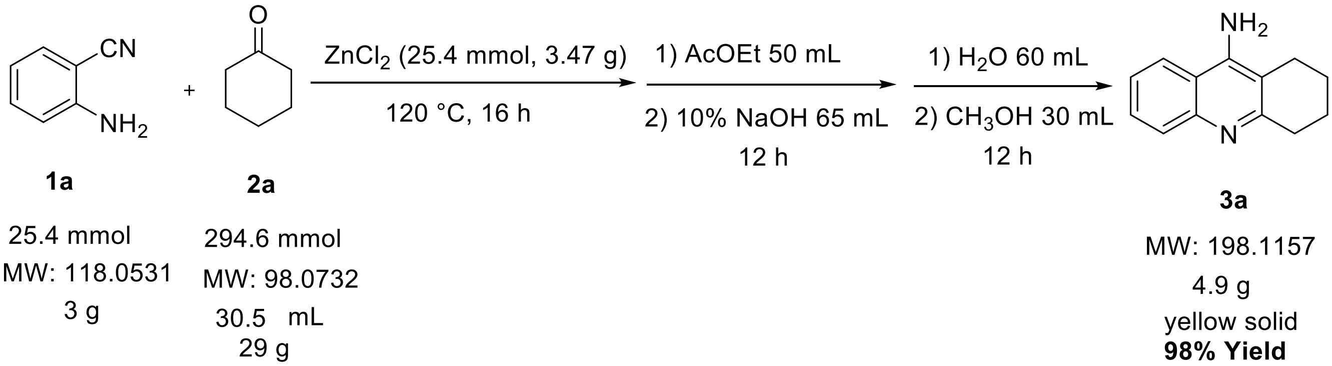

1a
25.4 mmol
MW: 118.0531
3 g

2a
294.6 mmol
MW: 98.0732
30.5 mL
29 g

3a
MW: 198.1157
4.9 g
yellow solid
98% Yield

Work-up: Light Green Flag.

Detailed Solvent: CH_3OH: problematic and hazardous. AcOEt and H_2O: recommended.

Eco-friendly synthesis of Tacrine 3a

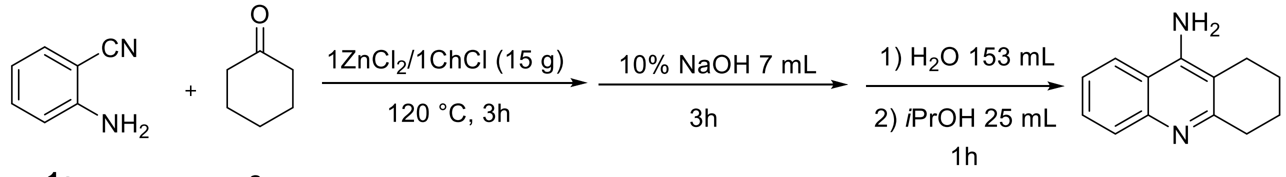

1a
25.4 mmol
MW: 118.0531
3 g

2a
25.4 mmol
MW: 98.0732
2.6 mL
2.5 g

3a
MW: 198.1157
4.9 g
yellow solid
98% Yield

Work-up: Green Flag.

Detailed Solvent: H_2O, *i*PrOH: recommended

AE

Classical synthesis of Tacrine 3a

M. W. Reactants: 118.0531 (**1a**) + 98.0732 (**2a**) = 216.1263

AE = 198.1157/216.1263 x 100 = 92%

Eco-friendly synthesis of Tacrine 3a

M. W. Reactants: 118.0531 (**1a**) + 98.0732 (**2a**) = 216.1263

AE =198.1157/216.1263 x 100 = 92%

RME

Classical synthesis of Tacrine 3a: 4.9 g/32.0 g [3.0 g (**1a**) + 29.0 g (**2a**)]x 100 = 15%

Eco-friendly synthesis of Tacrine 3a: 4.9 g/5.5 g [3 g (**1a**) + 2.5 g (**2a**)] x 100 = 89%

OE

Classical synthesis of Tacrine 3a: 15/92 x 100 = 16%

Eco-friendly synthesis of Tacrine 3a: 89/92 x 100 = 97%

EM

Classical synthesis of Tacrine 3a

Mass of non-benign reagents: 3g (**1a**)

EM = 4.9 g/[3 (**1a**) + 29 (**2a**) + 3.5 ($ZnCl_2$) + 23.8 (MeOH) = 59.3] g x 100 = 8.3%

Eco-friendly synthesis of Tacrine 3a

Mass of non-benign reagents: 3g (**1a**)

EM = 4.9 g /[3 (**1a**) + 2.5 (**2a**) + 8.0 ($ZnCl_2$) = 13.5] g x 100 = 36.3%

PMI

Classical synthesis of Tacrine 3a

Total amount of reactants: 3 g (**1a**) + 29 g (**2a**) + 3.47 g ($ZnCl_2$) = 35.47 g

Amount of the final product: 4.9 g

$PMI_{RXN}{}^{a}$ 35.47 g/4.9 g = 7.2 g g^{-1}

$PMI_{WU}{}^{b}$ 236.48 g/4.9 g = 48.3 g g^{-1}

3 g (**1a**) + 29 g (**2a**) + 3.47 g ($ZnCl_2$) + 45.1 g (AcOEt) + 72.15 g (NaOH) + 60 g (H_2O) + 23.76 g (MeOH) = 236.48 g

Eco-friendly synthesis of Tacrine 3a

Total amount of reactants: 3 g (**1a**) + 15 g (DES) + 2.5 g (**2a**) = 20.5 g

Amount of the final product: 4.9 g

$PMI_{RXN}{}^{a}$ = 20.5 g/4.9 g = 4.2 g g^{-1}

$PMI_{WU}{}^{b}$ = 97.92 g/4.9 g = 20.0 g g^{-1}

3 g (**1a**) + 2.5 g (**2a**) + 15 g (DES) + 7.77 g (NaOH) + 50 g (H_2O) + 19.65 g (*i*PrOH) = 97.92 g

[a] Process mass intensity $(PMI)_{RXN}$: chemicals and reaction solvents.

[b] Process mass intensity $(PMI)_{WU}$: chemicals and reaction solvents, solvents, and reagents in workup.

RI and RP

Classical synthesis of Tacrine 3a

Renewable sources: 60 g (H_2O)

RI: 60 g/4.9 g = 12.2 RP: 12.2/48.3 x 100 = 25.2%

Eco-friendly synthesis of Tacrine 3a

Renewable sources: 15 g (DES) + 50 g (H_2O) = 65 g

RI: 65 g/4.9 g = 13.3 RP: 13.3/20.0 x 100 = 66.5%

4. ^{1}H and ^{13}C NMR spectra

^{1}H NMR, 600 MHz, $CDCl_3$

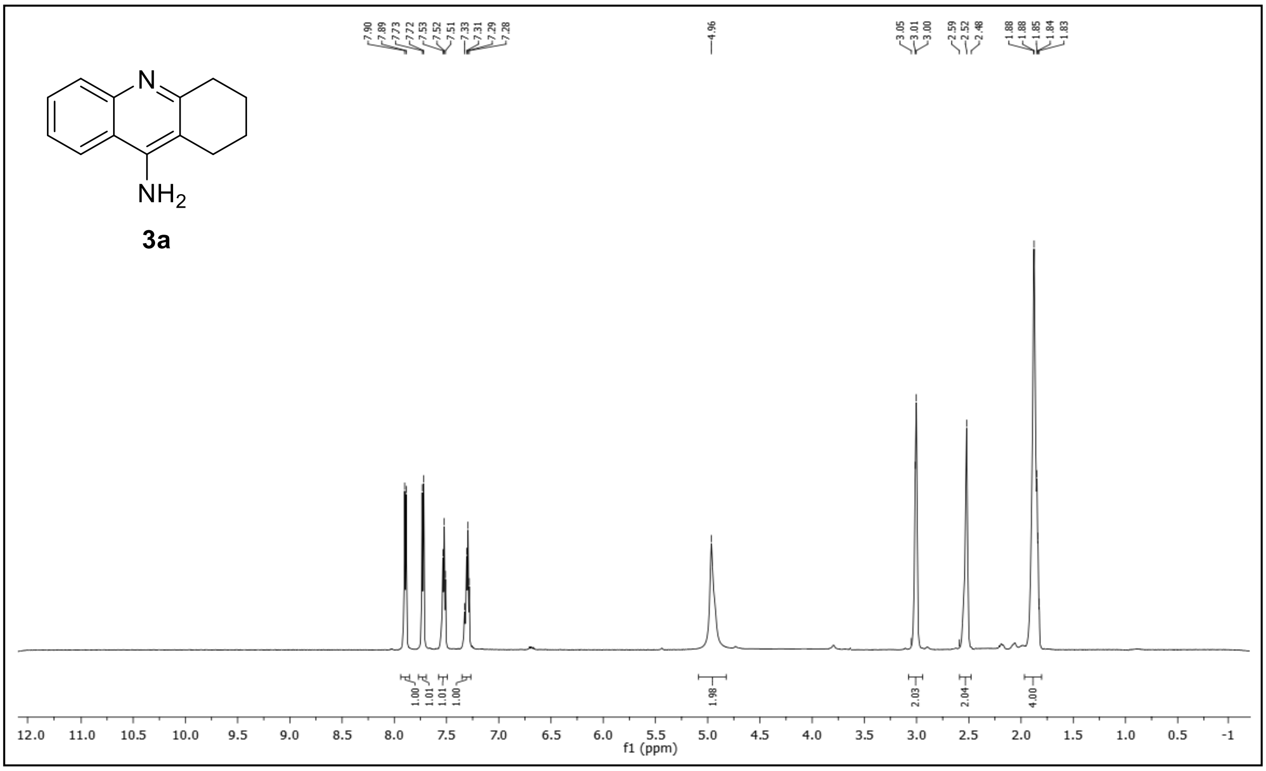

^{13}C NMR, 150 MHz, $CDCl_3$

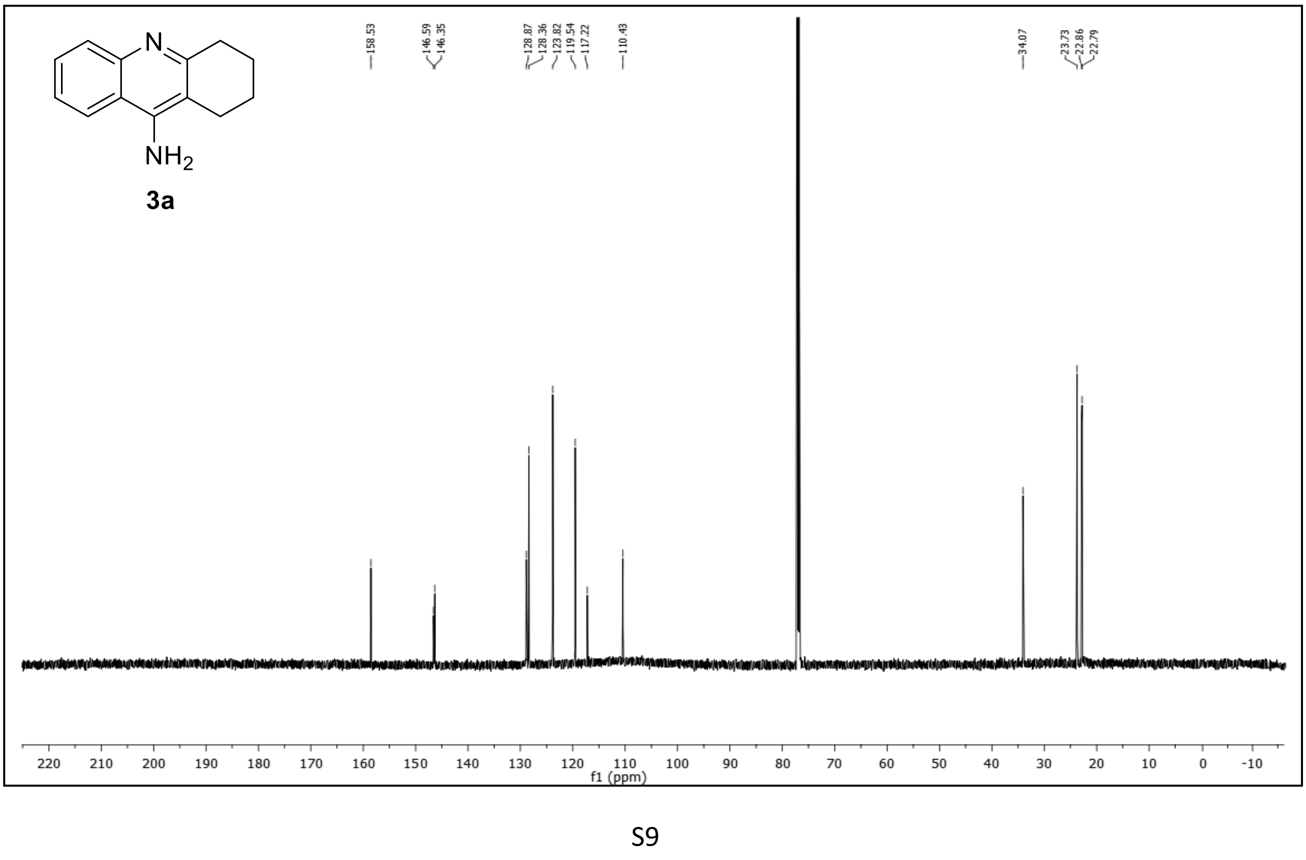

^1H NMR, 600 MHz, $CDCl_3$

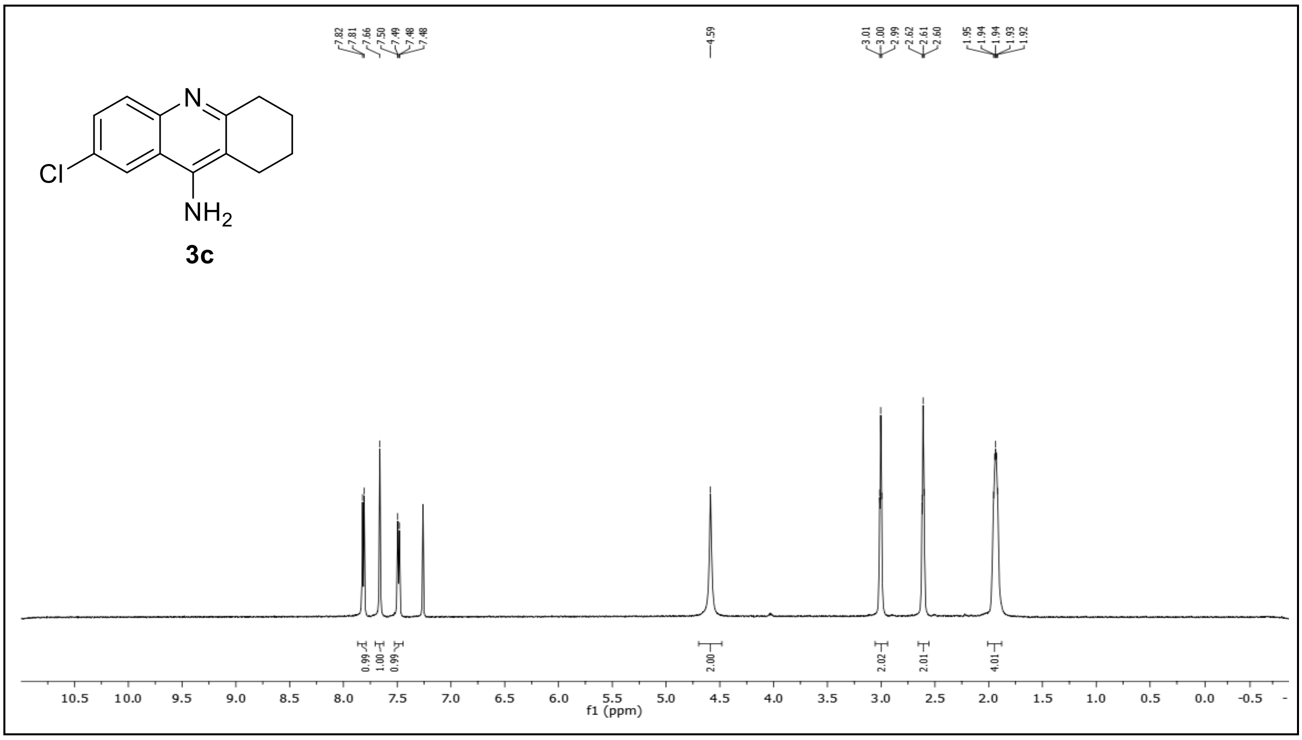

^{13}C NMR, 150 MHz, $CDCl_3$

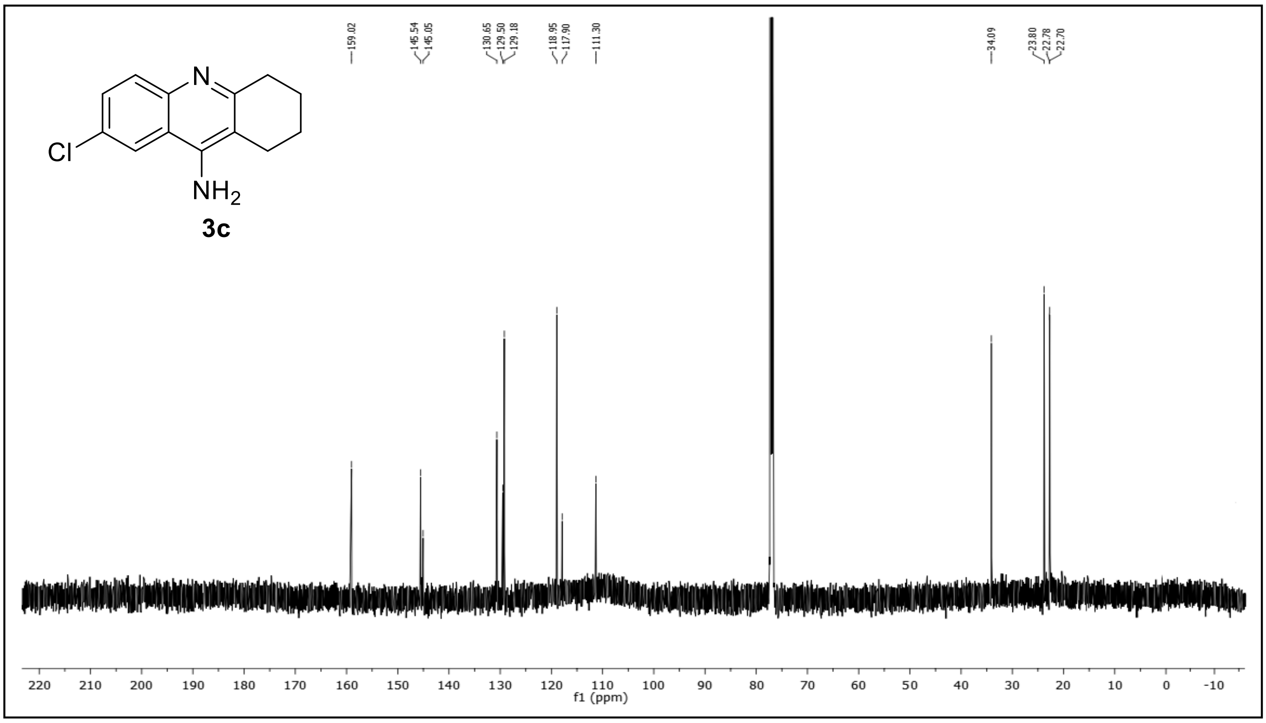

^{1}H NMR, 600 MHz, $CDCl_3$

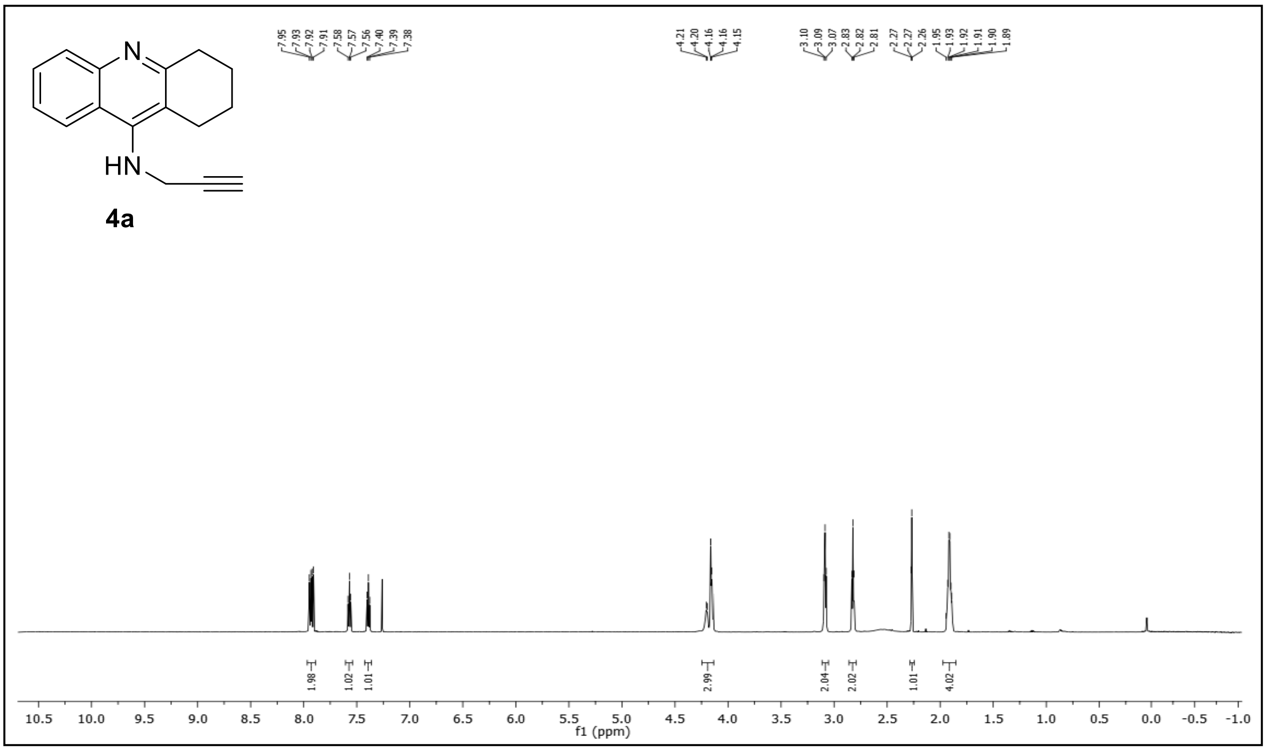

^{13}C NMR, 150 MHz, $CDCl_3$

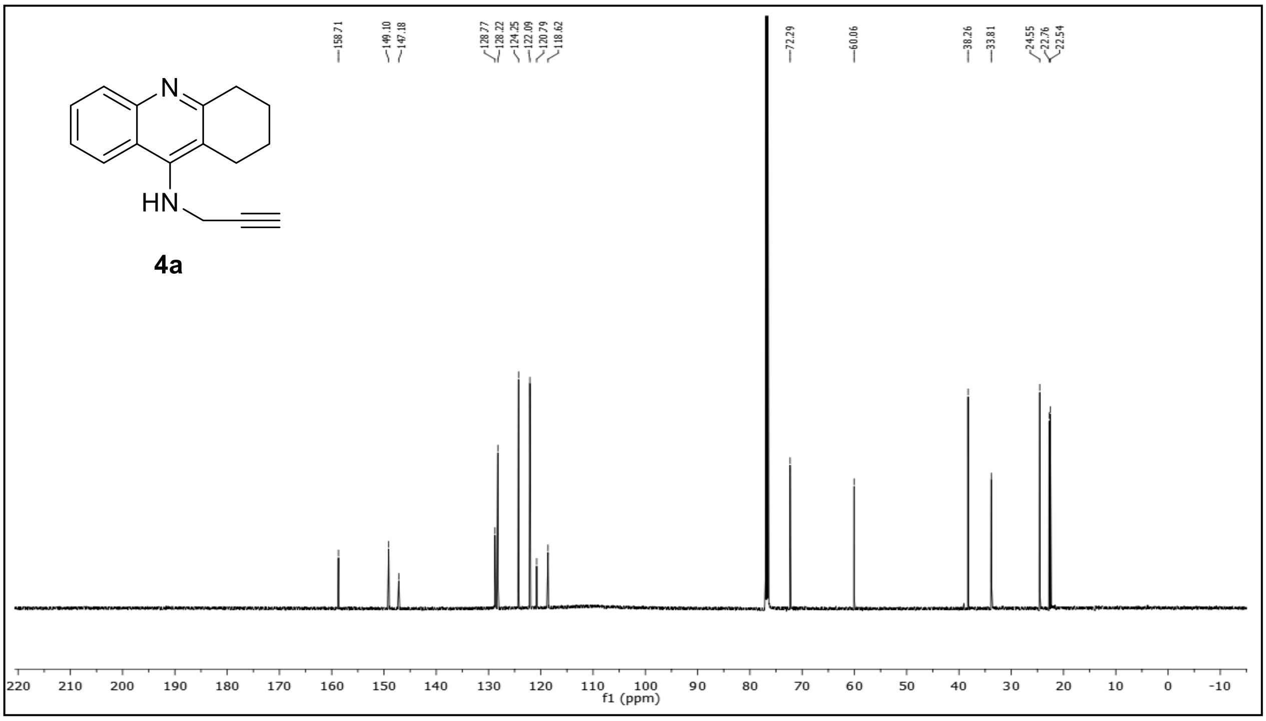

^{1}H NMR, 600 MHz, $CDCl_3$

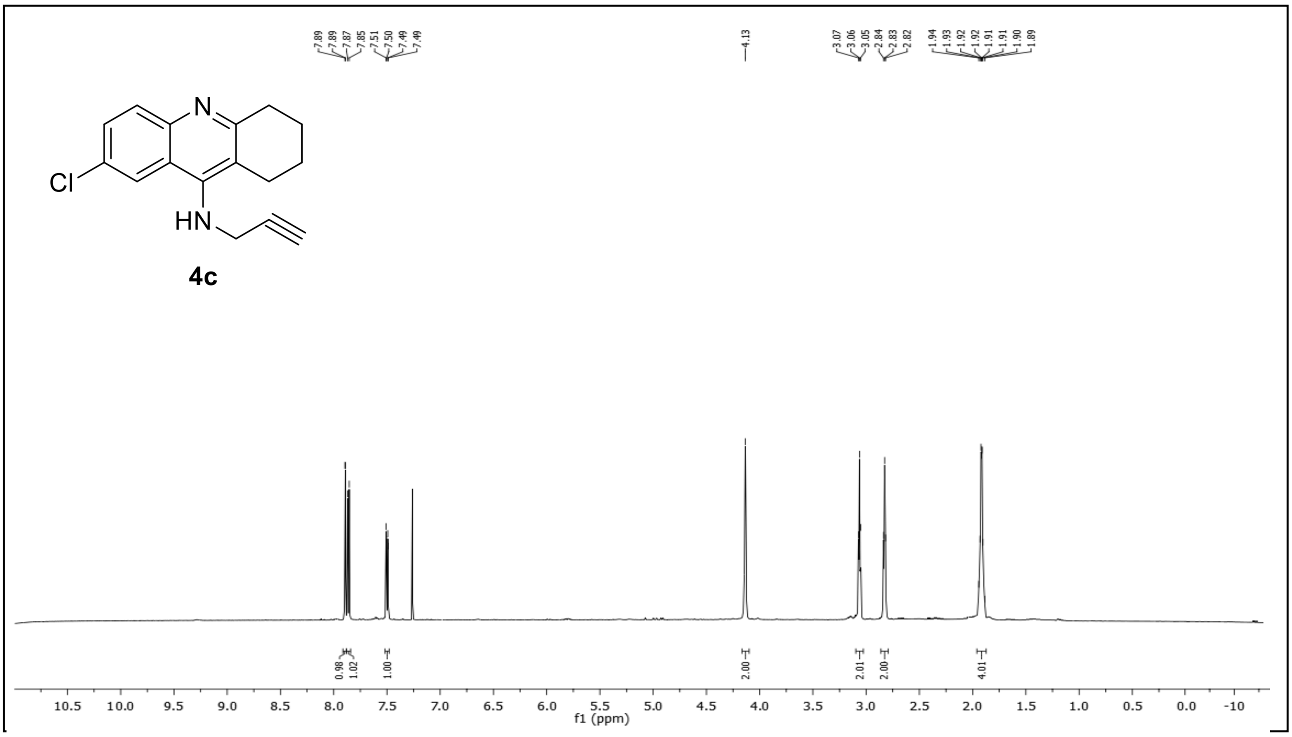

^{13}C NMR, 150 MHz, $CDCl_3$

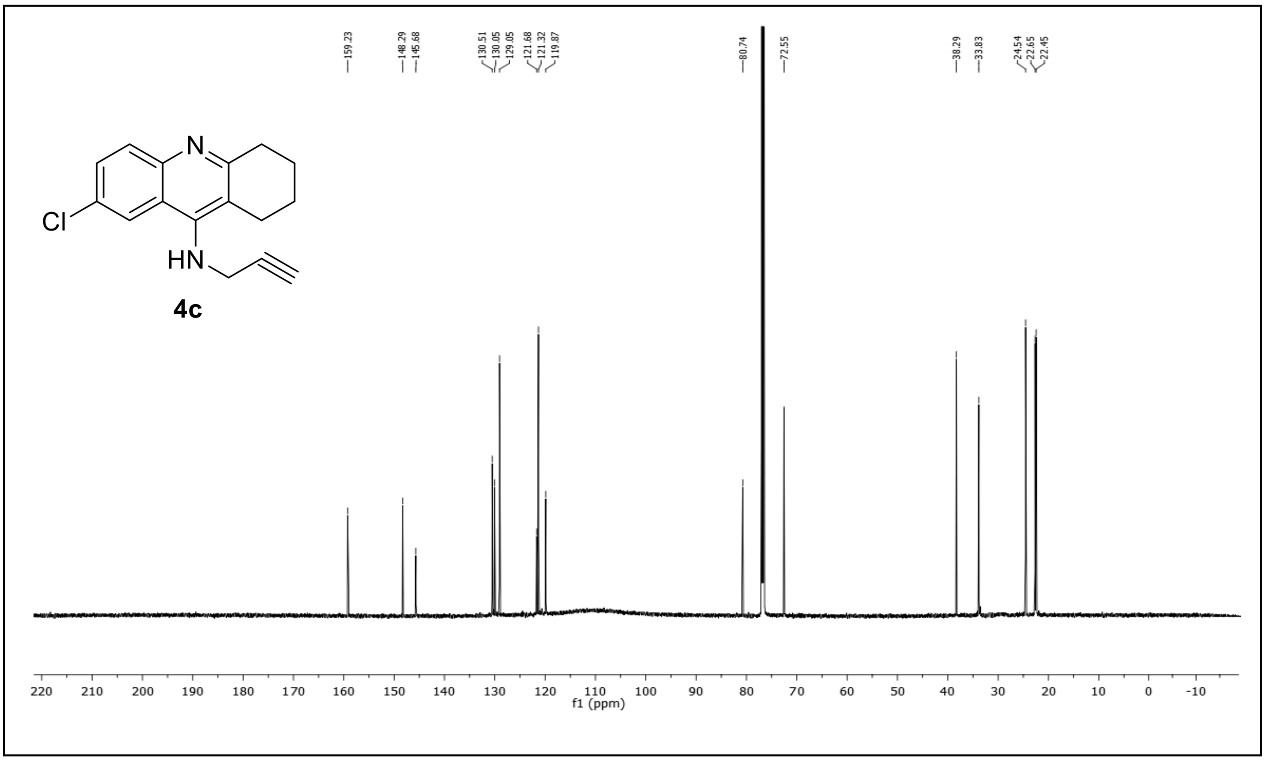

^{1}H NMR, 600 MHz, $CDCl_3$

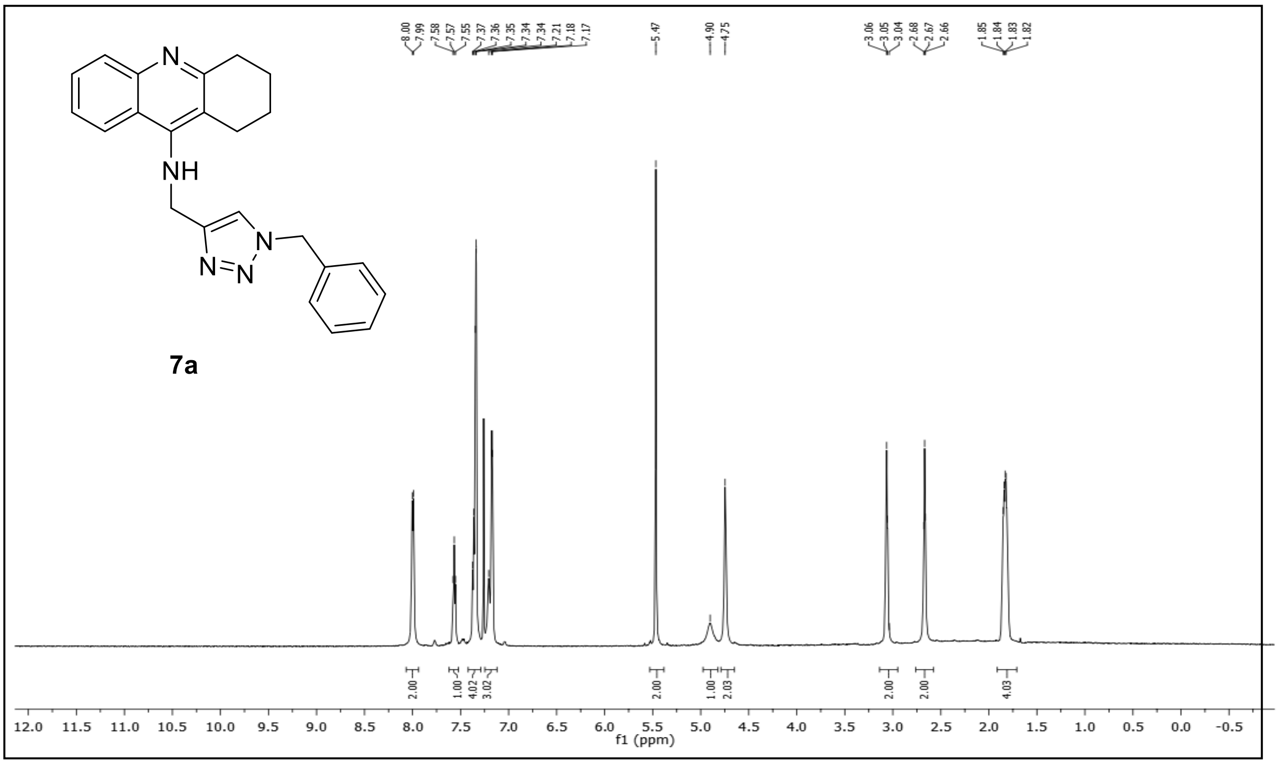

^{13}C NMR, 150 MHz, $CDCl_3$

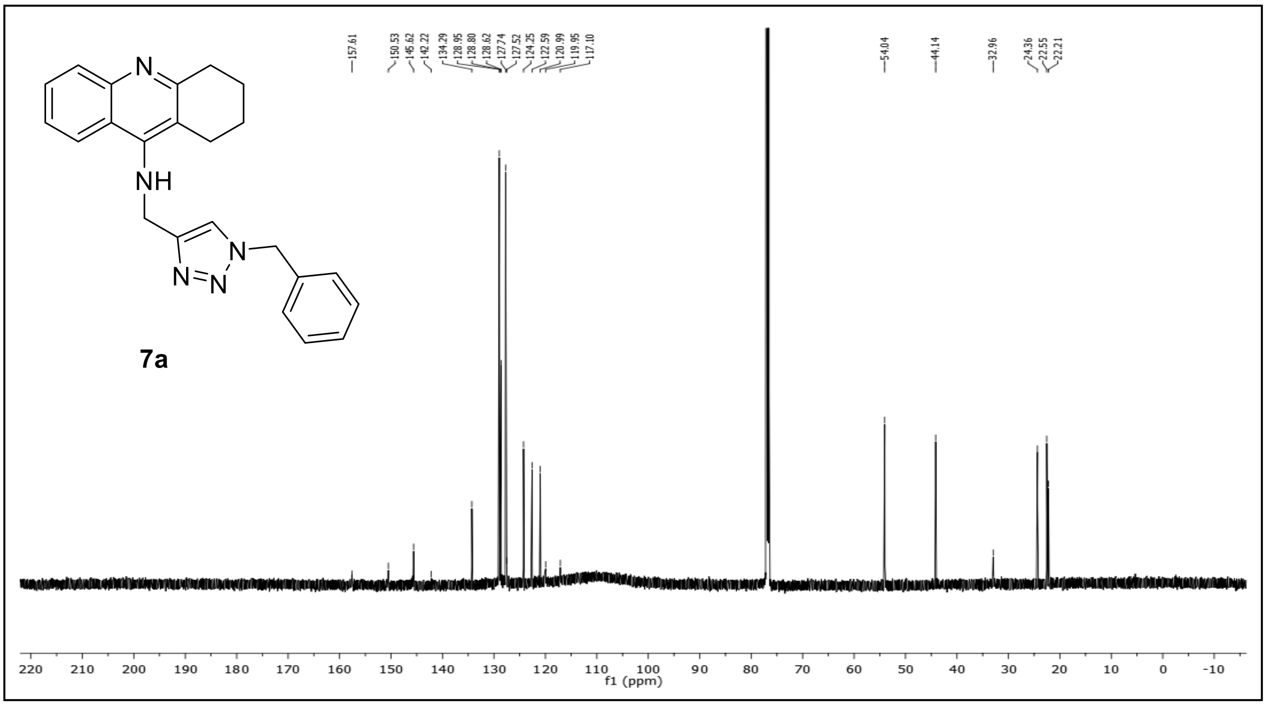

^{1}H NMR, 600 MHz, $CDCl_3$

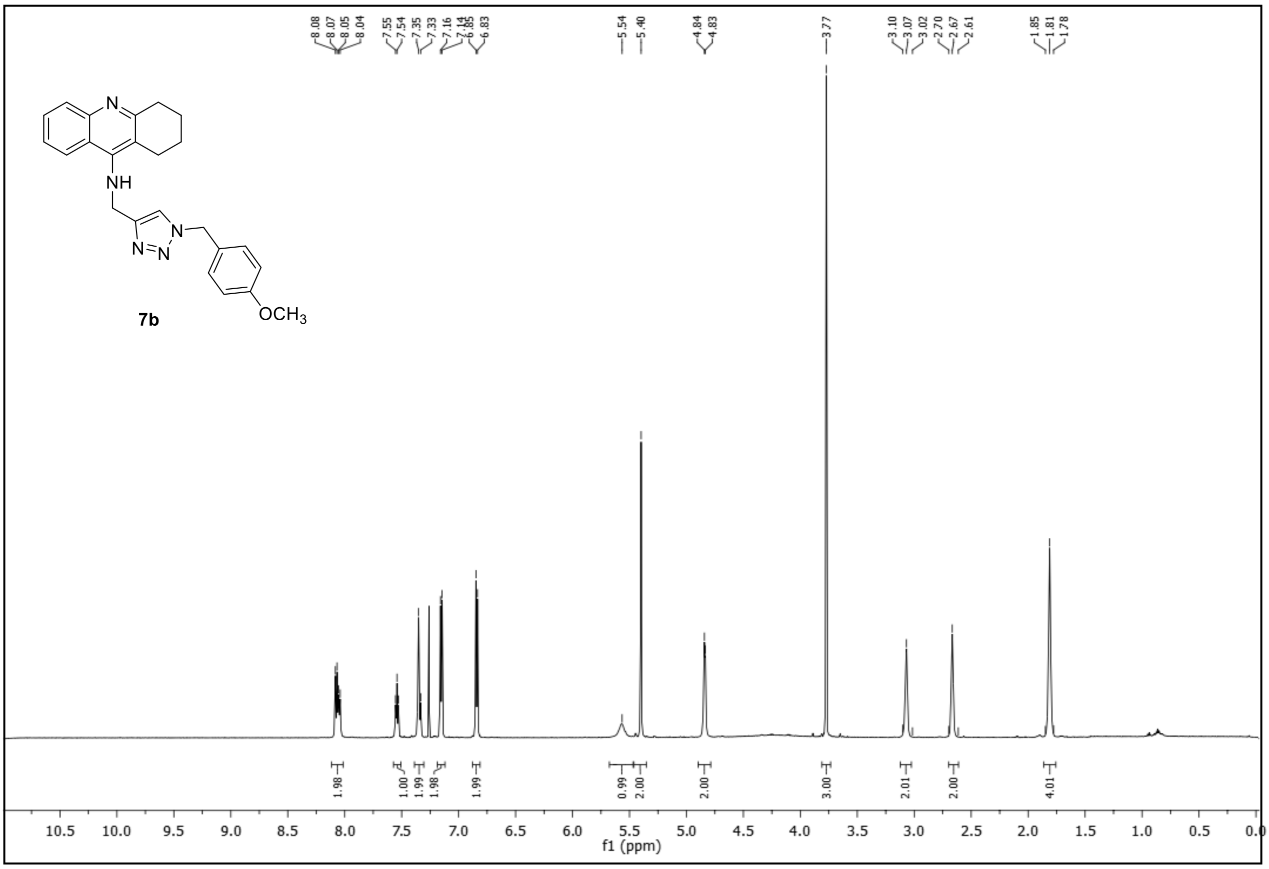

^{13}C NMR, 150 MHz, $CDCl_3$

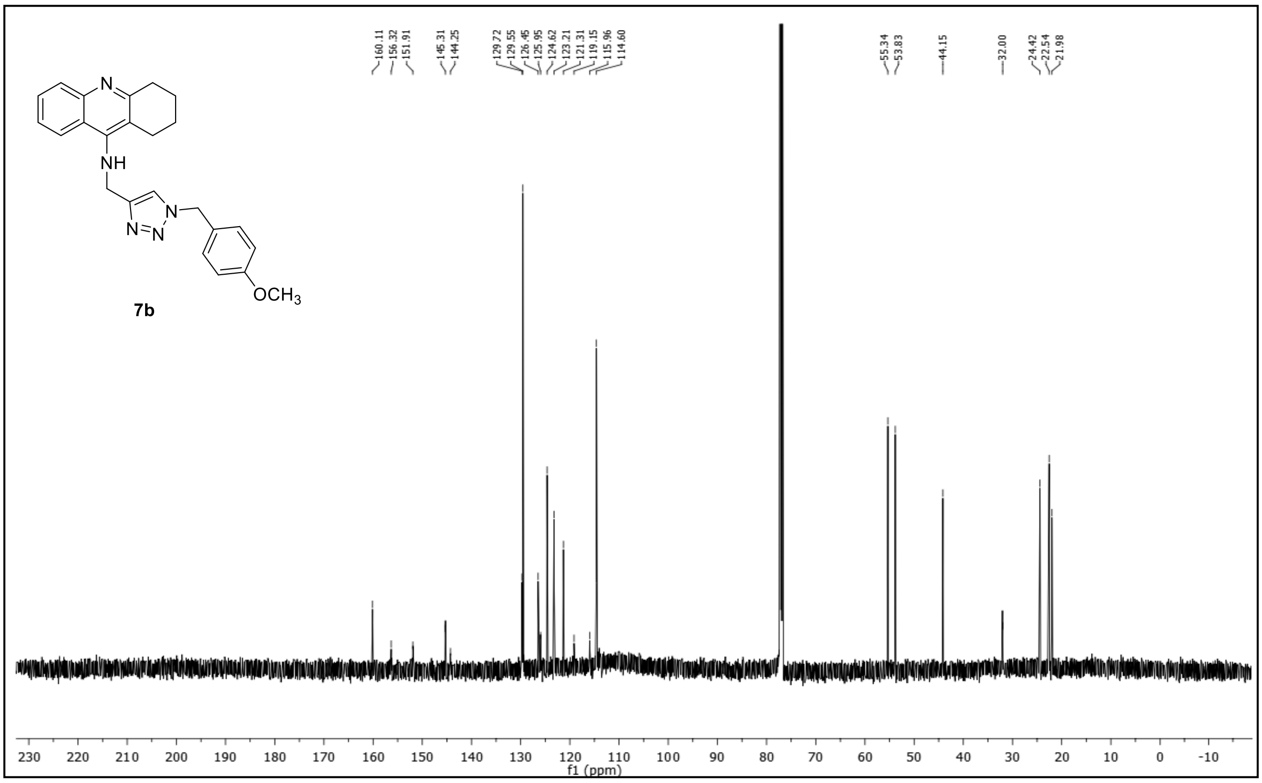

^{1}H NMR, 600 MHz, $CDCl_3$

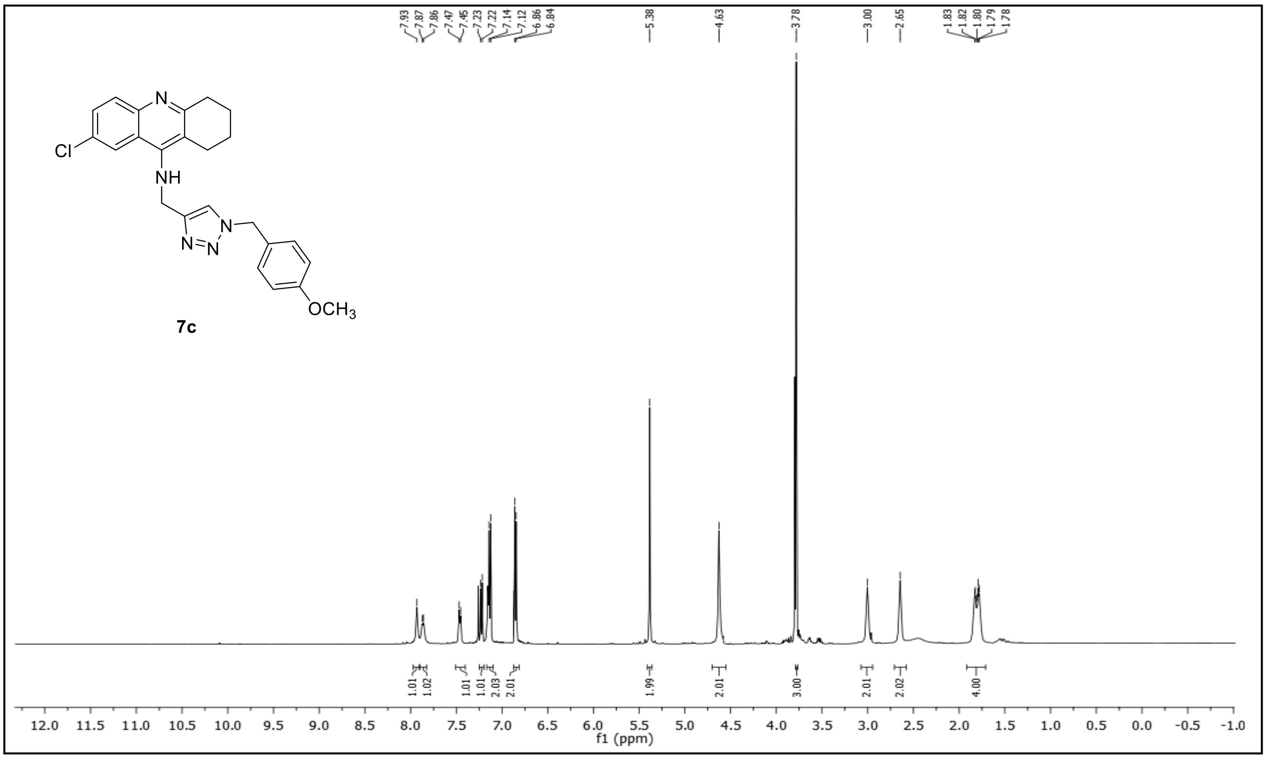

^{13}C NMR, 150 MHz, $CDCl_3$

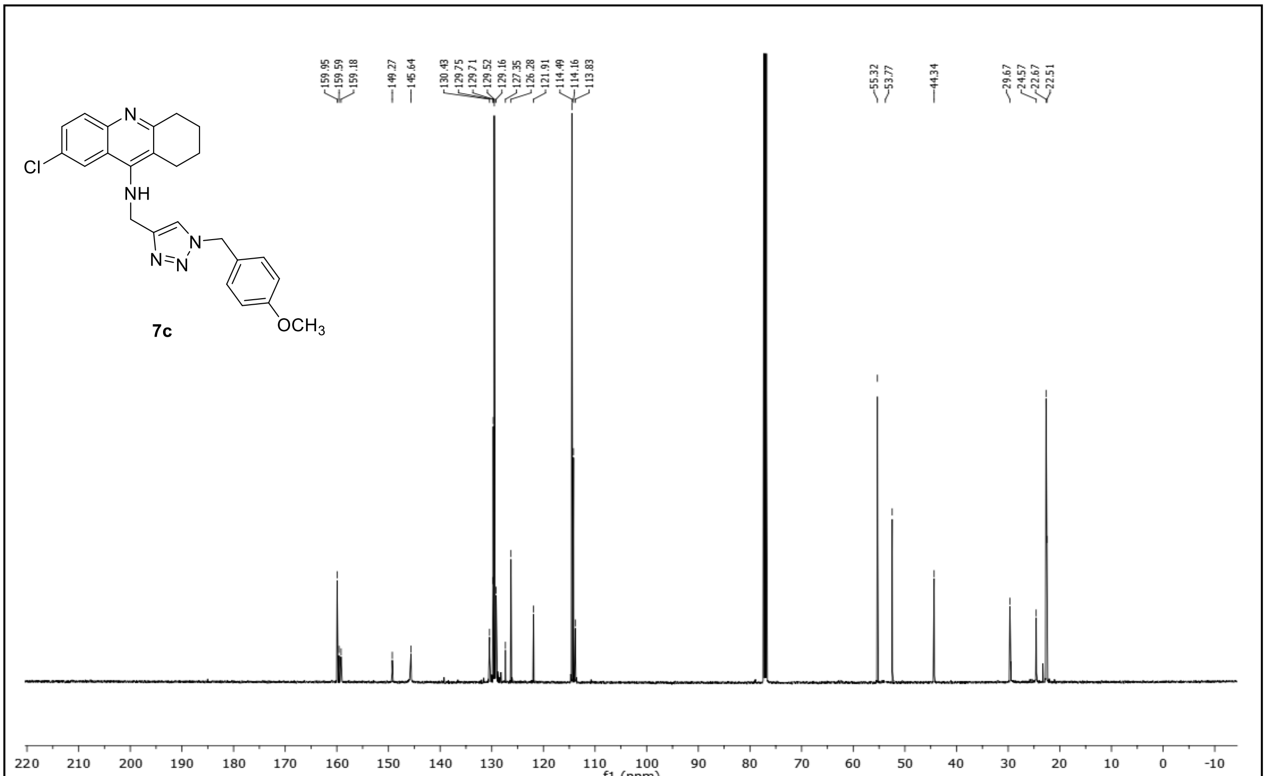

Supplement: Supplementary file 1 [file molecules-29-01399-s001.zip › molecules-2916196-supplementary.pdf]
